# Severe Dengue Epidemic, Sri Lanka, 2017

**DOI:** 10.3201/eid2604.190435

**Published:** 2020-04

**Authors:** Hasitha A. Tissera, Bernard D.W. Jayamanne, Rajendra Raut, Sakunthala M.D. Janaki, Yesim Tozan, Preshila C. Samaraweera, Prasad Liyanage, Azhar Ghouse, Chaturaka Rodrigo, Aravinda M. de Silva, Sumadhya D. Fernando

**Affiliations:** National Dengue Control Unit, Colombo, Sri Lanka (H.A. Tissera, B.D.W. Jayamanne, S.M.D. Janaki, P.C. Samaraweera);; Central Epidemiology Unit, Colombo (H.A. Tissera, A. Ghouse);; University of North Carolina, Chapel Hill, North Carolina, USA (R. Raut, A.M. de Silva);; New York University, New York, New York, USA (Y. Tozan);; Regional Directorate of Health Services, Kalutara, Sri Lanka (P. Liyanage);; University of New South Wales Sydney, Sydney, New South Wales, Australia (C. Rodrigo);; University of Colombo, Colombo (S.D. Fernando)

**Keywords:** Dengue, epidemic, Sri Lanka, surveillance, serotype, age group, viruses, vector-borne infections, mosquitoes, dengue virus

## Abstract

In 2017, a dengue epidemic of unexpected magnitude occurred in Sri Lanka. A total of 186,101 suspected cases and 440 dengue-related deaths occurred. We conducted a comprehensive analysis of this epidemic by comparing national surveillance data for 2017 with data from the preceding 5 years. In all Sri Lanka districts, dengue incidence in 2017 increased significantly over incidence during the previous 5 years. Older schoolchildren and young adults were more clinically symptomatic than those at extremes of age. Limited virologic surveillance showed the dominant circulating variant was dengue virus type 2 cosmopolitan genotype in the most affected district. One quarter of total annual cases were reported 5 weeks after the southwest monsoon started. Changes in vector abundance were not predictive of the increased incidence. Direct government expenditures on dengue control activities in 2017 were US $12.7 million. The lessons learned from this outbreak are useful for other tropical nations facing increasing dengue incidence.

Global incidence of dengue has increased 7-fold, from 8.3 million cases in 1990 to >58.4 million in 2013 (*1*). Currently, ≈390 million new infections occur annually in 128 dengue-endemic countries (*2*). Worldwide, ≈14,000–20,000 dengue-related deaths occur each year (*1*,*2*). In dengue-endemic countries, *Aedes* (*Stegomyia*) *aegypti* and *Ae.* (*Stegomyia*) *albopictus* mosquito vectors transmit the disease.

Sri Lanka, a tropical island in the Indian Ocean (population 21 million) (*3*), has reported dengue cases since the 1960s; seasonal epidemics predominantly affect areas that have annual rainfall >2,500 mm (*4*). However, until 1988, the more severe form of dengue virus (DENV) infection, dengue hemorrhagic fever, was reported only sporadically (*5*,*6*). During 1991–2008, dengue epidemics occurred once every few years on the background of endemic transmission (*6*). A disproportionate epidemic occurred in 2009 comprising 35,008 suspected cases (incidence 170 cases/100,000 population) and 346 deaths (case-fatality rate 1%) (*7*). During 2010–2016, dengue became a major public health problem; cases increased steadily (from 28,473 in 2011 to 55,150 in 2016) throughout the country but disproportionately affected the most populated Western province (*7*). In 2017, a total of 186,101 suspected cases and 440 dengue-related deaths were reported to the Central Epidemiology Unit of the Ministry of Health, Sri Lanka (*7*). This number is the highest number of suspected cases reported in a single calendar year in Sri Lanka since dengue was designated a notifiable disease in 1996. We compared the temporal, epidemiologic, virologic, entomologic, and climatic characteristics of the 2017 dengue epidemic with those of the epidemics during the preceding 5 years (2012–2016).

## Methods

### Data Sources

#### Epidemiology

We obtained epidemiologic data from the integrated national communicable disease surveillance system, which captures symptomatic dengue patients classified according to a standard case definition based on the 1997–2011 World Health Organization classification (*8*). Etiologic screening was conducted with NS1 antigen testing or dengue antibody assays. However, given the limited diagnostic test availability, especially in remote areas of the country, many cases were clinically diagnosed using the surveillance case definition (*9*). We based the population data we used on the last national census before the outbreak (2012) and projections for the following years (*10*).

#### Virology

The Central Epidemiologic Unit has an established ongoing fever surveillance cohort of 500 families in the catchment areas of the National Institute of Infectious Diseases, the main referral hospital for dengue patients in the most affected Colombo district. Viruses isolated from blood samples of patients suspected to have dengue collected during December 2016–December 2017 were sequenced (Sanger technique) at the University of North Carolina (Chapel Hill, NC, USA; Appendix). The sequences were aligned using Sequencher Software version 5.4.6 (https://www.genecodes.com) and phylogenetics analysis was performed on MEGA X software (*11*).

#### Entomology

Systematic entomologic surveillance is conducted in all districts of the country according to the guidelines issued by the National Dengue Control Unit of Sri Lanka’s Ministry of Health. This surveillance comprises routine cluster surveys (systematic sampling of 100 premises per block) conducted monthly by designated entomologists. The presence of *Ae. aegypti* and *Ae. albopictus* larvae and pupa are reported as Breteau index, Container index, and Premise index/House index (*12*). A premise or container was considered positive if a single larva or pupa was found in a receptacle within its boundaries, both indoors and outdoors.

#### Meteorology

We extracted daily rainfall and temperature data during 2012–2017 from the Department of Meteorology for each district. The weekly cumulative rainfall in millimeters and mean temperature (°C) were calculated using daily observations. We obtained Oceanic Niño Index data as a measure of El Niño Southern Oscillation activity from the US National Oceanic and Atmospheric Administration (https://www.noaa.gov). The Oceanic Niño Index tracks the 3-month average sea surface temperatures in the east-central tropical Pacific from 120° to 170° West (Niño 3.4 region) (*13*). We correlated meteorologic parameters with the dengue incidence in 2017 and of the preceding 5 years for Colombo district (Western province, wet zone; annual rainfall >2,500 mm) and Jaffna district (Northern province, dry zone; annual rainfall 1,250–2,500 mm). We selected these 2 districts because both maintained a high dengue incidence during the 6 years examined and are representative of Sri Lanka’s 2 contrasting climatic zones. Colombo district, in the wet zone, receives most of its rainfall from the southwest monsoon during May–September. Jaffna district, in the dry/semiarid zone, receives most of its annual rainfall from the northeast monsoons during December–February.

#### Cost Estimates

We estimated the direct costs of dengue control and outbreak response during epidemic year 2017 from the Ministry of Health perspective. Direct costs were costs of routine dengue control activities and costs of outbreak response activities. More specifically, routine dengue control activities included standard measures of premise inspections and removal of vector breeding sites, larval control, entomologic surveillance, development of electronic databases, health education, advertising through media, purchases for integrated vector control (fogging machines and chemicals), and operating costs of administrative offices. Outbreak response activities (after the epidemic) were establishment of extra high-dependency units in public hospitals, door-to-door premise inspection and source reduction programs in high-risk districts through civil–military cooperation, deployment of vector control brigades in high-risk districts, and extra-duty allowance for public health staff during outbreak months (May–August). We extracted the financial costs from the National Dengue Control Unit annual budget and financial expenditure records. For public health staff not exclusively engaged in dengue-related activities, we calculated the amounts on a pro rata basis according to the approximate number of hours spent on dengue-related work. This cost evaluation did not include in-kind costs, such as uncompensated work hours by community members or other stakeholders, because of the unavailability of records. We extracted all costs in local currency and converted to US dollars using the annual official exchange rate of the Sri Lanka rupee (LKR) 152.5 to US $1 in 2017 (World Bank, https://data.worldbank.org/indicator/PA.NUS.FCRF?end=2016&locations=LK&start=1960&view=chart).

### Statistical Analyses

We analyzed the data using SPSS Statistics 21.0 (IBM, https://www.ibm.com). Descriptive statistics are shown as frequencies, means (± SD), and proportions. We examined the statistical significance of population proportions (count data) and mean differences (continuous data) by z-score test and used χ^2^ test for dichotomous data with statistical significance set at 0.05. The effect size is expressed as odds ratios or relative risk with 95% CIs. We used a distributed lag nonlinear model (*14*) for the analysis of exposure-lag response association between weather variables and dengue incidence. We conducted distributed lag nonlinear model construction and lagged correlation analysis in the R statistical environment (*15*).

### Sequence Availability and Ethics Clearance

We deposited the DENV-2 sequences used in phylogenetic analysis in GenBank (accession nos. MK579857–61. The Ethics Review Committee of the Faculty of Medicine, University of Colombo (EC-18-004) provided ethics clearance for this study.

## Results

**Figure 1:**
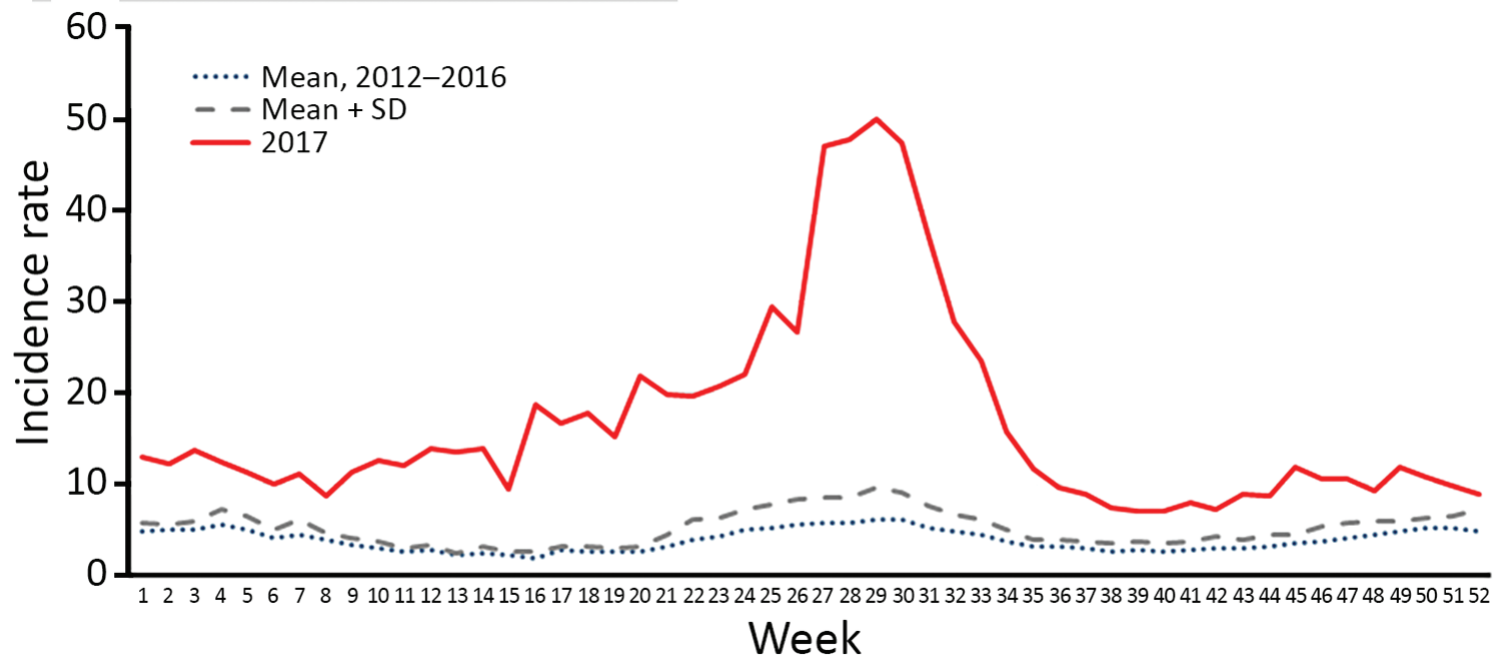
Comparison of the weekly mean attack rates of dengue reported in 2017 with the 5-year historical mean (2012–2016), Sri Lanka. Rates are cases per 100,000 population. Source: (*7*).

In 2017, a total of 186,101 suspected dengue cases (866 cases/100,000 population) and 440 dengue-related deaths (case-fatality rate 0.24%) were reported to the national surveillance system. The weekly average of reported dengue cases was significantly higher than the average for the preceding 5 years (3,570 vs. 792 cases/week, p<0.0001, greater than mean for 2012–2016 + 2 SD) throughout the year. The national surveillance picked up an unusual increase in cases in the northern and eastern parts of the country from the latter half of 2016. However, a much larger increase in incidence was notable from week 16 of 2017 and peaked at week 29 when 10,699 cases were reported. Approximately one quarter of total dengue cases in 2017 were reported during weeks 27–31 (Figure 1).

### Spatial Distribution of Dengue Incidence

**Figure 2:**
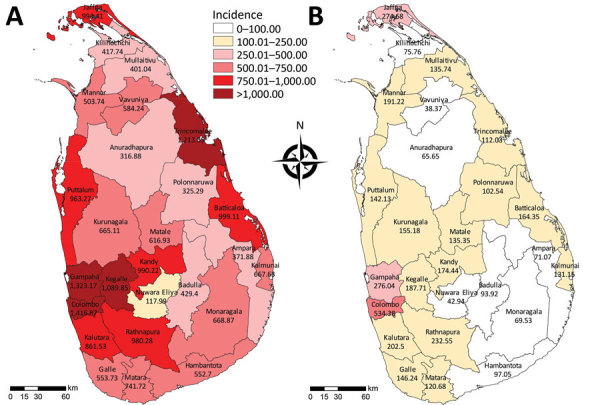
Comparison of dengue incidence rates per district in 2017 with the 5-year (2012–2016) average, Sri Lanka. A) Incidence rate in 2017. B) Historical mean incidence rate during 2012–2016. Incidence is cases per 100,000 population. Source: (*7*).

Sri Lanka’s 9 provinces are divided into 25 administrative districts (Table 1; Figure 2). In 2017, the most dengue cases were reported from the 2 most populous Colombo and Gampaha districts within the Western province (Colombo, 1,419/100,000; Gampaha, 1,323/100,000). Three other districts also had an incidence rate >1,000 cases/100,000 population, and 10 districts reported rates above the national average (Table 1). The lowest incidence rate (118/100,000 population) was reported from the Nuwara-Eliya district, which is into Central province at 1,800 m above sea level. In all districts, dengue incidence in 2017 was significantly higher than the average incidence in the preceding 5 years (Table 1). When we repeated the comparison against each of the preceding 5 years (instead of the average), the difference remained significant for all districts.

**Table 1:** Incidence of dengue, Sri Lanka*

Province, administrative district	Mean incidence†		
	2017	2012–2016	Fold increase in 2017‡
Western			
Colombo	1,419	503	2.8
Gampaha	1,323	252	5.2
Kalutara	861	190	4.5
Eastern			
Trincomalee	1,214	107	11.4
Batticaloa	1,001	157	6.4
Kalmunai	698	131	5.3
Sabaragamuwa			
Kegalle	1,090	174	6.3
Ratnapura	978	214	4.6
Northern			
Jaffna	996	251	4.0
Vavuniya	583	87	6.7
Mannar	500	175	2.9
Kilinochchi	418	70	6.0
Mulativu	402	130	3.1
Central			
Kandy	990	162	6.1
Matale	616	125	4.9
Nuwara Eliya	118	40	2.9
Southern			
Matara	976	89	10.9
Galle	553	138	4.0
Hambantota	419	107	3.9
North-western			
Puttalam	965	119	8.1
Kurunegala	665	135	4.9
Uva			
Moneragala	668	64	10.4
Badulla	430	88	4.9
North-Central			
Polonnaruwa	325	96	3.4
Anuradhapura	317	60	5.3
National average	866	189	4.6

*Source: (*7*). †Cases/100,000 population. ‡Increases were significant in all districts (p<0.05) compared with the average for 2012–2016.

### Demographic Profile

**Figure 3:**
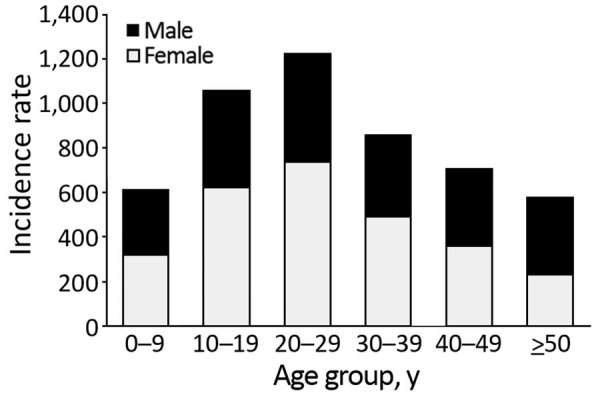
Dengue incidence rates by age group and sex, Sri Lanka, 2017. Incidence is cases per 100,000 population. Source (*7*).

The sex distribution of dengue patients in 2017 was similar (99,464 [55.2%] male, 80,724 [44.8%] female; p>0.05). The mean age of dengue patients nationwide was 29.7 years, but age varied widely by province (Table 2). Mean age was significantly lower than the national average (p<0.001) in Eastern (22.3 years), Northern (27.6 years), and Western (28.7 years) provinces and significantly higher in Sabaragamuwa (36.5 years), North Central (34.1 years), and Southern (33.2 years) provinces (Table 2). Incidence was highest for persons 20–29 years of age (1,225 cases/100,000 population), followed by persons 10–19 years (1,057/100,000 population). Persons >50 years of age were least affected (580/100,000 population) (Figure 3). Age-specific incidence rates among provinces differed noticeably. For example, young children (5–9 years) were the most affected group in the Eastern province (Appendix Table 3).

**Table 2:** Age distribution of dengue patients, Sri Lanka, 2017*

Province	Mean age, y	+ SD	25th percentile	75th percentile
Western	28.7†	18.5	14.0	40.0
Eastern	22.3†	16.4	9.0	31.0
Sabaragamuwa	35.7‡	18.1	22.0	49.0
Northern	27.6†	16.5	17.0	36.0
Central	29.5	17.6	16.0	41.0
Southern	33.2‡	17.3	21.0	45.0
North-Western	31.1	18.3	17.0	44.0
Uva	31.1	16.9	19.0	42.0
North-Central	34.1‡	14.3	23.0	43.0
National average	29.7	18.2	16.0	42.0

*Source: (*7*). †Significantly lower mean age than the national average, p<0.05. ‡Significantly higher mean age than the national average, p<0.05.

**Table 3:** Type and proportion of breeding habitats positive for *Aedes aegypti* mosquitoes, Sri Lanka, 2017*

Province	Discarded items, %	Water storage containers and tanks, %	Ponds and ornamental items, %	Wells and tube wells, %	Natural water collections, %	Other miscellaneous items, %†
Western	50.9	10.0	3.7	1.6	2.2	31.5
Eastern	33.9	22.9	3.0	11.5	1.6	27.0
Sabaragamuwa	55.6	5.1	5.1	9.5	0.8	24.0
Northern	18.8	55.0	2.9	0.2	0.1	23.0
Central	21.9	42.9	1.8	0.0	0.2	33.2
Southern	41.6	23.7	4.8	0.0	4.1	25.9
North-Western	46.4	21.7	1.1	5.6	0.2	24.9
Uva	41.2	39.2	1.2	0.0	0.5	17.9
North-Central	19.1	30.1	8.1	0.0	0.0	42.8
National average	38.7	23.9	3.4	5.8	1.3	26.8

*Total number of premises inspected: 279,728; total *Ae. aegypti*–positive containers: 9,699. Source: (*7*). †Including refrigerator trays, nonfunctional cisterns, pet feeding cups, gutters, concrete slabs, and any other water-collecting containers.

### Phylogenetic Analysis

**Figure 4:**
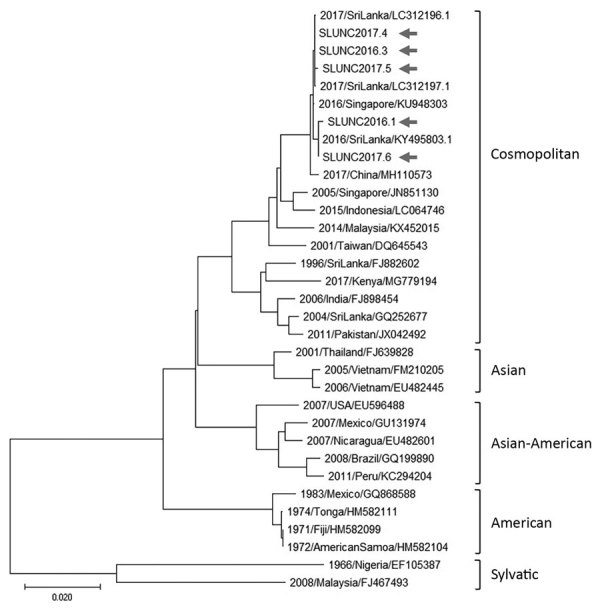
Phylogenetic tree for 5 dengue virus 2 (DENV-2) isolates from late 2016 and 2017 dengue epidemic (arrows), Sri Lanka, and reference DENV-2 strains. The tree is based on a 1,485-nt fragment that encodes the envelope protein. Classification and naming of DENV-2 genotypes are based on (*16*). The evolutionary history was inferred using the neighbor-joining method (*17*). The optimal tree with the sum of branch length = 0.44012906 is shown. The tree is drawn to scale, with branch lengths in the same units as those of the evolutionary distances used to infer the phylogenetic tree. The evolutionary distances were computed using the maximum composite likelihood method (*18*) and are in the units of the number of base substitutions per site. This analysis involved 33-nt sequences. All ambiguous positions were removed for each sequence pair (pairwise deletion option). The final dataset comprised 1,485 positions. Evolutionary analyses were conducted in MEGA X (*12*). Scale bar indicates nucleotide substitutions per site.

From blood samples collected during December 2016–December 2017 from 140 persons with acute fever, a total of 44 (31%) persons were positive for DENV RNA (DENV-2, 39; DENV-1, 4; DENV-2/3, 1). Of these, 5 DENV-2 isolates were cultured and sequenced (PrM-E region). A neighbor-joining phylogenetic tree (Figure 4) made with a mix of other DENV-2 genotype references placed the new sequences within the cosmopolitan genotype (*17*,*19*). However, this strain differed from other DENV-2 cosmopolitan strains reported previously in Sri Lanka (*18*,*20*) and was more closely related to variants isolated from Singapore and China during 2014–2017.

### Entomologic Surveillance

The mean national Breteau index for 2017 was similar to that for 2013–2016 (11.67% vs. 12.9%; p = 0.833). Provincial-level differences were not significant. Types and proportions of water-retaining containers positive for *Ae. aegypti* mosquitoes varied by province (Table 3). In Western, Sabaragamuwa, and North-Western provinces, discarded water-holding containers were the most common receptacles positive for larvae. In contrast, in Central and Northern provinces, which had an extended drought in 2017, vector breeding was observed mainly in water storage containers and tanks. However, entomologic parameters did not correlate with the disproportionate increase in dengue cases in 2017.

### Climatic Factors

**Figure 5:**
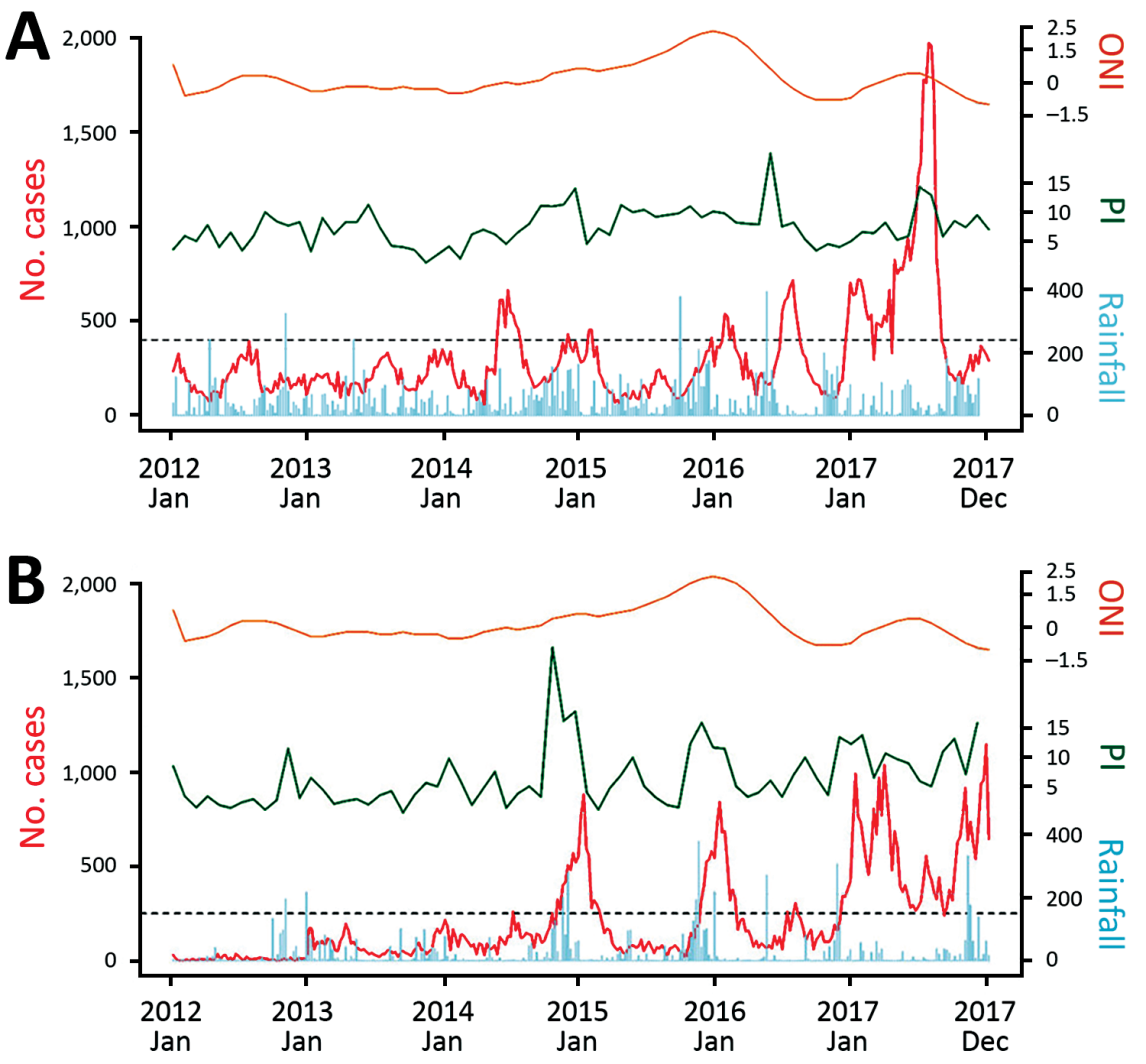
Time series plot showing weekly number of reported dengue cases (red line), ONI (orange line), PI (green line), and weekly cumulative rainfall, mm (blue line), Sri Lanka, 2012–2017. A) Colombo district. B) Jaffna district. ONI, Oceanic Niño Index; PI, Premise Index.

We assessed whether dengue incidence correlated with climatic parameters (Figure 5). The mean annual rainfall in Colombo district was lower in 2017 than in 2012–2016 (1,908 mm vs. 2,447 mm). In the Jaffna district, cumulative rainfall during 2017 (1,342 mm) was similar to the average annual rainfall for 2012–2016 (1,297 mm). El Niño conditions (Oceanic Niño Index >0.5) were observed in 2015 and 2016 but not in 2017. In addition, temperature patterns in both districts during 2017 and during the preceding 5 years did not change notably. Therefore, weather conditions were stable in both districts throughout 2012–2017, despite the disproportionate increase in dengue incidence in 2017.

### Cost Analysis

We estimated the direct cost of dengue control and outbreak response activities during the epidemic year of 2017 to be US $12.7 million (LKR 1.938 billion), corresponding to a per capita cost of US $0.64 (Table 4). Of this total, US $4.4 million (35%; US $0.22 per capita) was spent on outbreak response activities.

**Table 4:** Direct costs of dengue control and outbreak response activities, Sri Lanka, 2017*

Type of activity	Sri Lanka rupee	US $
Routine dengue control activities		
Personnel: public health staff salaries	1,060,340,000	6,955,512
Development of health education material and advertising	5,000,000	32,798
Implementation of dengue awareness campaign through electronic and print media	10,000,000	65,597
Integrated vector management; insecticides and fogging machines	156,000,000	1,023,313
Recurrent costs at National Dengue Control Unit	7,000,000	45,918
Routine vector control and recurrent costs in periphery	25,000,000	163,992
Subtotal	1,263,340,000	8,287,131
Outbreak response activities		
Personnel: public health staff extra duty pay for outbreak response, at national and regional/district levels	176,723,333	1,159,252
Outbreak response brigades: brigade staff salaries and other expenses during outbreak response	114,000,000	747,806
Purchase of mini cabs for vector control (50 cabs)	75,000,000	491,977
Establishment of high-dependency units in public hospitals	181,000,000	1,187,306
Intensified door-to-door premise inspection campaign in high-risk districts	128,000,000	839,642
Subtotal	674,723,333	4,425,983
Total	1,938,063,333	12,713,114
Cost per capita	96.90	0.64

*Source: (*7*).

## Discussion

In 2017, a dengue epidemic in Sri Lanka resulted in an unprecedented number of cases and dengue-related deaths and a considerable strain on resources in terms of direct expenditures. Incidence increased substantially throughout the country; even the district with the lowest incidence for 2017 had a 3-fold increase over the average of the preceding 5 years. The epidemic primarily affected older schoolchildren and young adults in the workforce, with some marked disparities in age-specific incidence rates across provinces. Entomologic and climatic factors (except rainfall) did not explain the increased incidence. Limited virologic surveillance during 2017 in the district most affected (Colombo) showed DENV-2 dominance.

Temporal trends in dengue incidence are closely linked to the demographic characteristics of Sri Lanka. Historically, seasonal dengue outbreaks have heavily affected the country’s urban areas and, more conspicuously, the Colombo district (which comprises 11% of the country’s population and has the highest population density) (*10*). In entomologic surveys, more than half of *Ae. aegypti* vector breeding occurred in discarded items (e.g., containers, tires) in the Western province, signifying the effect of poor container waste management, which is conducive for vector breeding. On a global scale, the high vector-to-host ratio profoundly affects dengue epidemiology (*21*–*25*). In Vietnam, a study of 2 outbreaks during 2005–2008 showed that dengue incidence increased in a critical window of population density (3,000–7,000/km^2^) and declined thereafter (*26*).

In most Sri Lanka provinces, including the Western province, the highest dengue incidence rate occurred among persons 20–29 years of age (without any sex difference), followed by persons 10–19 years of age. These age groups comprise children attending secondary schools (up to 18 years of age), students of tertiary education institutions, and young working adults. The same trend has been observed in the Southeast Asia region, where older schoolchildren have a higher risk for clinical illness (*27*). However, the dengue incidence in younger age groups (0–9 years) might be underestimated because of asymptomatic or mild primary infection. Approximately half of primary dengue infections in children are inapparent (*28*,*29*), whereas primary infections in adults more often result in overt disease (*30*). In Sri Lanka, incidence also decreased among elderly persons, which cannot be explained by asymptomatic infections. According to Sri Lanka’s 2012 census, 31.3% of the population was 10–29 years of age, and only 12.4% was >60 years of age (*10*). Comparable data from other countries in Asia give conflicting results about age-specific dengue incidence. For example, in Taiwan and Singapore during 2010–2015, the prevalence of dengue was higher in persons >60 years of age than in younger persons, a plausible reflection of an aging but active elderly population (*31*–*33*). On the other hand, in countries such as India, Vietnam, and Brazil, dengue incidence is increasing in a higher proportion of persons in younger age groups (*27*,*34*,*35*).

In many countries, serotype switches of circulating dengue viruses have been associated with severe epidemics when the population is exposed to a new serotype (*36*,*37*). Host immunity against 1 dengue serotype only partially protects against other serotypes. In fact, heterotypic antibodies may exaggerate the inflammatory response during infection with a different serotype (*38*). Circulating serotypes in Sri Lanka were monitored in the past by a handful of research studies in the absence of a national virologic surveillance program. A study conducted during 2003–2006 (*39*) and another during a large epidemic in 2009 (*40*) concluded that DENV-1 was the dominant circulating serotype in all these periodic epidemics. This observation remained unchanged in 2 subsequent epidemics in 2010 and 2012 (*41*). However, during 2003–2006, DENV-2 and DENV-3 serotypes caused between-epidemic background dengue transmission (*39*). After 2010, the number of DENV-1 cases progressively increased, taking over the background transmission from DENV-2 infections, which might have led to a loss of immunity to DENV-2 in the population. Limited virologic surveillance from the Colombo district demonstrated DENV-2 dominance in the 2017 samples, but this finding cannot be extrapolated to the entire country. The viruses isolated from the 5 patients had almost identical sequences and belonged to the cosmopolitan genotype of DENV-2. The phylogenetic analysis demonstrates this strain to be a new strain (for Sri Lanka) that most likely came from Southeast Asia or China (*18*).

Of all the climatic factors studied, only monsoon rainfall had a clear relationship over the years with the dengue incidence. Historically, the incidence of dengue in Sri Lanka shows 2 peaks per year, each of which occurs a few weeks after onset of the 2 monsoons affecting the country. The southwest monsoon brings in heavy rainfall midyear (May–September) to Western, Sabaragamuwa, Southern, and Central provinces, and the northeast monsoon brings a relatively heavy rainfall during December–February for the Northern, North Central, Uva, and Eastern provinces. The midyear peak in dengue incidence in 2017 was exceptionally high in all provinces affected by the southwest monsoon (weeks 25–35).

The increase in dengue incidence in 2017 could not be explained by entomologic parameters because they did not differ significantly between preceding years and 2017. The types of breeding sites reported in each of the provinces varied but also did not have any relationship with the reported case incidence.

Sri Lanka’s government-led dengue control activities mainly focus on vector control. These methods include routine measures of integrated vector control that are in place periodically throughout the year and outbreak control measures during epidemics: health education, source reduction of vector breeding sites, chemical fogging, and punitive measures (e.g., fines, warnings for harboring mosquito breeding sites). Integrating a biological control method into this framework by introducing the larvicidal *Bacillus thuringiensis* bacterium did not yield promising results in a recently concluded field trial in the city of Colombo and its suburbs in the Western province (*42*). Outbreak control measures usually are conducted as civilian–military partnerships focusing on breeding site removal. These methods come into effect only when an epidemic is ongoing and are not helpful in preventing one. Government expenditure on dengue control activities (excluding costs of curative services and indirect costs) is substantial, and 35% of the cost was on outbreak control, which could have been averted by better outbreak prevention. During the epidemic year of 2012, per capita spending for dengue in Colombo district was US $0.42 (US $0.48 when adjusted for depreciation of the LKR during 2012–2017) (*43*). In 2017, per capita expenditure was US $0.64 in a countrywide cost assessment.

Our findings are subject to several limitations. A large volume of the national surveillance system data originates from the public health services and in-ward facilities of private hospitals. Data from outpatient departments of private hospitals and general practitioners are underrepresented. In addition, facilities for laboratory diagnosis are limited. Although most cases were reported as suspected cases diagnosed with syndromic definition using clinical criteria and serial blood counts, on-demand rapid antigen detection tests are becoming increasingly available. The number of dengue infections in Sri Lanka still can be underestimated because clinically inapparent infections are not counted. Because of the lack of national virologic surveillance, genomic data on infecting viruses are available only from the Western province, which might not be representative for the rest of the country.

In retrospect, the key early indicators of the disproportionate epidemic in 2017 were the increase in cases in the latter half of 2016 and the gradual transition of the circulating strain (limited evidence from 1 district). An increase in the number of cases every year after monsoon rains is clear in all years studied. The direct costs of dengue control are huge once an outbreak occurs and could have been better spent on outbreak prevention rather than on outbreak control if prevention activities were sustained throughout the intermonsoon period and intensified during the monsoon rains. Epidemiologic surveillance based on suspected cases (vs. confirmed cases) is not ideal but is the best many dengue-endemic countries can do with limited access to point-of-care diagnostic testing. Capturing data from currently underreporting healthcare providers (e.g., general practitioners) is a priority to get an accurate picture of dengue in Sri Lanka. Establishing a virologic surveillance system covering the entire country is important and is becoming feasible as new third-generation sequencing technologies become portable and less expensive. However, health education that results in behavior change of communities remains the most effective way of reducing vector breeding sites to prevent outbreaks. The government should facilitate health education as well as a good waste management policy backed by periodic audits.

Appendix Additional methods for analysis of a severe dengue epidemic, Sri Lanka, 2017.
