# Supplementary material for: Severe Dengue Epidemic, Sri Lanka, 2017
Source: Emerg Infect Dis. 2020 Apr;26(4):682–91. doi: 10.3201/eid2604.190435 (PMC7101108; doi:10.3201/eid2604.190435)
Supplement: Appendix — Additional methods for analysis of a severe dengue epidemic, Sri Lanka, 2017. [file 19-0435-Techapp-s1.pdf]

Article DOI: https://doi.org/10.3201/eid2604.190435

Severe Dengue Epidemic, Sri Lanka, 2017

Appendix

Supplementary Methods

Viral Isolation and Sequencing from Blood Samples

Virus culture was done by inoculating 15 μL of PCR-positive samples and 185 μL GIBCO Dulbecco's Modified Eagle Medium: Nutrient Mixture F-12 (DMEM-F-12) supplemented with sodium bicarbonate containing 2% fetal bovine serum (FBS) on C6/36 mosquito cells at 37°C and 5% CO_2. Viral RNA was isolated from C6/36 culture supernatants using QIAamp Viral RNA Mini Kit from Qiagen using manufacturer's protocol. Complementary DNA (cDNA) was made using isolated RNA as a template and SuperScript® III Reverse transcription from Invitrogen. cDNA was amplified by using NEB Phusion High Fidelity kit and primers spanning precursor membrane (prM) and envelope (E) proteins. The final product was isolated by gel electrophoresis and sequenced by Sanger technique.

Appendix Table. Age-specific dengue incidence by province, Sri Lanka*

Province	Age-specific incidence per 100,000 population											
	0–4 y	5–9 y	10–14 y	15–19 y	20–24 y	25–29 y	30–34 y	35–39 y	40–44 y	44–49 y	50–64 y	≥65 y
Western	1,050.58	1,742.60	2,181.65	2,011.07	1,896.41	1401.98	1,562.64	1,247.91	1,137.35	1,029.01	994.95	849.80
Eastern	807.18	1,332.27	1,285.31	1,143.92	705.01	512.37	516.14	390.49	320.15	148.27	280.80	160.95
Sabaraga-muwa	245.22	366.83	644.99	889.44	1,107.81	949.64	1,159.29	962.81	958.22	854.35	904.92	714.39
Northern	327.35	496.99	542.26	877.08	843.38	552.60	510.08	400.41	285.90	172.67	260.59	240.18
Central	303.90	591.12	999.58	1,262.67	893.26	671.94	724.10	613.29	587.65	564.96	522.86	366.98
Southern	178.39	269.14	461.82	724.48	994.49	702.40	723.89	614.68	562.29	467.06	551.51	380.82
North Western	291.63	486.48	632.97	690.28	636.56	497.07	564.06	496.52	440.14	378.89	444.78	302.17
Uva	56.65	120.35	166.88	308.57	308.01	231.03	208.88	177.74	164.36	93.92	149.93	95.77
North Central	23.38	30.54	66.18	217.98	391.58	318.48	333.14	269.94	237.15	233.63	160.05	76.96
Total	500.76	833.17	1,080.28	1,151.98	1,094.62	819.80	899.87	735.29	672.90	586.71	608.32	472.82

*Source: sentinel site surveillance
